# Comparison of different preparation techniques of dried blood spot quality controls in newborn screening for congenital adrenal hyperplasia

**DOI:** 10.1371/journal.pone.0252091

**Published:** 2021-05-20

**Authors:** Nóra Grecsó, Anita Zádori, Ákos Baráth, Zsolt Galla, Gábor Rácz, Csaba Bereczki, Péter Monostori

**Affiliations:** Metabolic and Newborn Screening Laboratory, Department of Pediatrics, University of Szeged, Szeged, Hungary; University of Pisa, ITALY

## Abstract

In newborn screening, samples suspected for congenital adrenal hyperplasia (CAH), a potentially lethal inborn error of steroid biosynthesis, need to be confirmed using liquid chromatography–tandem mass spectrometry. Daily quality controls (QCs) for the 2^nd^-tier CAH assay are not commercially available and are therefore generally prepared within the laboratory. For the first time, we aimed to compare five different QC preparation approaches used in routine diagnostics for CAH on the concentrations of cortisol, 21-deoxycortisol, 11-deoxycortisol, 4-androstenedione and 17-hydroxyprogesterone in dried blood spots. The techniques from Prep1 to Prep5 were tested at two analyte concentrations by spiking aliquots of a steroid-depleted blood, derived from washed erythrocyte suspension and steroid-depleted serum. The preparation processes differed in the sequence of the preparation steps and whether freeze-thaw cycles were used to facilitate blood homogeneity. The five types of dried blood spot QCs were assayed and quantitated in duplicate on five different days using a single calibration row per day. Inter-assay variations less than 15% and concentrations within ±15% of the nominal values were considered acceptable. Results obtained by means of the four dried blood spot QC preparation techniques (Prep1, Prep2, Prep4 and Prep5) were statistically similar and remained within the ±15% ranges in terms of both reproducibility and nominal values. However, concentration results for Prep3 (spiking prior to three freeze-thaw cycles) were significantly lower than the nominal values in this setting, with differences exceeding the ±15% range in many cases despite acceptable inter-assay variations. These findings have implications for the in-house preparation of QC samples in laboratory developed tests for CAH, including 2^nd^-tier assays in newborn screening.

## Introduction

Congenital adrenal hyperplasia (CAH; OMIM 201910, OMIM 202010) is a severe inborn disorder of steroid biosynthesis [1, 2]. CAH is characterized by cortisol insufficiency and accumulation of other androgenic steroid precursors that cause androgen excess in both sexes, significantly reducing life quality [1, 2]. Moreover, a disturbed sodium homeostasis with hyponatremia and hyperkalemia in certain forms of CAH can result in a hyponatremic shock and death in early infancy [2].

Newborn screening (NBS) of CAH, performed worldwide including in 24 European countries and all states of the USA, has markedly improved the diagnosis and outcome of this possibly lethal disorder [1–3]. Here, dried blood spots (DBSs) are used for the measurement of 17-hydroxyprogesterone (17OHP) by means of a fluorimetric immunoassay [1, 2]. The relatively high number of false-positives in the 1^st^-tier testing necessitates the confirmation of CAH [2]. Second-tier testing using liquid chromatography–tandem mass spectrometry (LC-MS/MS) is recommended [1, 2, 4], which uses the same DBS specimen as in the primary screening, together with daily quality control (QC) samples for quality assurance purposes [5–9].

However, daily QCs for cortisol (Cort), 21-deoxycortisol (21Deox), 11-deoxycortisol (11Deox), 4-androstenedione (4AD) and 17OHP are currently commercially unavailable. Even if the Centers for Disease Control and Prevention (CDC) does provide 2^nd^-tier CAH testing QC material, these samples are not intended for daily quality assurance purposes according to the CDC [10]. Thus, laboratories performing in-house methods for CAH must generally prepare daily QCs themselves. The preparation technique (e.g. sample collection, non-matrix spiking volume, homogeneity) can greatly affect the quality of the DBS sample which, in turn, is important for reliable quantitation and diagnosis [11–13]. However, despite its widespread use and diagnostic importance, DBS preparation in NBS is not covered in recommendations for other applications of DBS such as therapeutic drug monitoring [11–13].

Previously published approaches for the preparation of home-made QCs for CAH differed in the sequence of the preparation steps and whether homogeneity of the blood was supported with freeze-thaw cycles [6–9, 14]. Techniques included application of the spiked blood onto filter paper with [8, 9] or without [6] prior freezing and a consequent hemolysis of the blood to improve homogeneity; three freeze-thaw cycles of the steroid-depleted whole blood, followed by spiking [7]; or two weeks of freezing [14].

Until now, no reports were available on the comparison of the various QC preparation procedures used in the routine diagnostics for Cort, 21Deox, 11Deox, 4AD and 17OHP in DBS. Thus, in addition to a technique [6] similar to general protocols reported for non-NBS applications [11–13], we set out to test additional QC preparation approaches that are also used in the routine diagnostics for CAH [7–9, 14] in terms of inter-assay reproducibility and agreement with the nominal concentrations.

## Materials and methods

### Reagents

Deuterated internal standards (ISs) D_4_-Cort, D_8_-21Deox, D_2_-11Deox, D_5_-4AD and D_8_-17OHP were purchased from Cambridge Isotope Laboratories (Andover, MA, USA); unlabeled Cort, 21Deox, 11Deox, 4AD and 17OHP from Sigma-Aldrich (St. Louis, MO, USA); methanol (LC-MS grade) and formic acid (LC-MS grade) from Honeywell (Charlotte, NC, USA); and acetonitrile (LC-MS grade) from Merck (Darmstadt, Germany). Ultrapure water (18.2 MΩ.cm), filtered through a 0.22 μm pore size membrane, was obtained from a Merck Millipore Direct-Q 3 UV system (Billerica, MA, USA).

### Preparation of the DBS samples

Heparinized blood from a single healthy volunteer (author P.M.) was centrifuged at 1000 x g for 15 min. Pooling blood from more than one volunteer was not necessary as the total volume of blood needed for the study was relatively low. Additionally, using blood from a single person can help eliminate potential incompatibility issues caused by the small amount of residual plasma that may remain after washing the red blood cells [15]. The erythrocytes were washed three times with phosphate-buffered saline; the absence of hemolysis was confirmed after each step (plasma and supernatant were discarded). The washed erythrocytes were mixed stepwise with commercially available steroid-depleted serum (BBI Solutions, Crumlin, UK) from a single bottle. Hematocrit values were repeatedly measured after each step by means of a Beckman Coulter UniCel DxH 600 hematology analyzer (Beckman Coulter, Inc, Brea, CA, USA) until the target hematocrit of approx. 50% was reached [15], corresponding to the average hematocrit in newborns [11]. The steroid-depleted blood was split into five aliquots. Subsequent steps for the five different QC preparation procedures are summarized in Table 1.

**Table 1:** Description of the five QC preparation procedures.

	Common preliminary preparation steps from Prep1 to Prep5	Preparation-specific procedures on aliquots of the steroid-depleted blood	Common final preparation steps from Prep1 to Prep5
**Prep1**	1. Heparinized red blood cells washed 3 times with phosphate-buffered saline 2. Steroid-depleted serum added stepwise to achieve a hematocrit of 50% 3. Steroid-depleted blood split into 5 aliquots	Spiking without any freeze-thaw cycles	1. Spiked blood mixed gently but thoroughly 2. Spiked blood applied onto filter paper 3. Samples dried at room temperature for up to 24 h 4. Samples stored in sealed aluminium bags with silica gel desiccants at -70°C
**Prep2**		Three freeze-thaw cycles, followed by spiking	
**Prep3**		Spiking, then 3 freeze-thaw cycles	
**Prep4**		Spiking, then 2 weeks storage at -70°C	
**Prep5**		Two weeks storage at -70°C, followed by spiking	

Spiking of the five aliquots from the same steroid-depleted blood was performed using equal volumes of spiking solutions in saline (20 μl solution added to 1980 μl steroid-depleted blood, corresponding to a 1% non-matrix spike volume, i.e. a 100-fold dilution of the analytes). The samples were then mixed gently but thoroughly by applying repeated manual inversion of the tubes containing spiked blood immediately after spiking and then in every 2-3 min for 30 min. Final concentrations of the QCs were 30(60) and 90(180) nM for steroids (Cort), respectively. Thereafter, samples were applied (70 μl per spot) by a single person onto Ahlstrom-Munksjö TFN filter paper cards (Ahlstrom-Munksjö Germany GmbH, Bärenstein, Germany) using a manual air displacement pipette and dried at room temperature for up to 24 h. A sample volume of 70 μl per circle was chosen because this volume allowed to punch four 4.7 mm spots from each blood circle in an „X” shape. Here, all punches cover similar peripheral locations within a circle, which can improve the reproducibility of the measurements and allows for more efficient utilization of the prepared QCs. All QCs (and the DBS calibrators) were stored in sealed aluminium bags with silica gel desiccants at -70°C.

The five types of QCs were assayed in duplicates on five different days together with a single daily calibration row which was then used to calculate concentrations of all QCs on that day. Measurements took place approximately once per week within 1 month. Inter-assay variations <15% and concentration results within ±15% of the nominal values were considered acceptable in line with recommendations [16].

All procedures followed were in accordance with the ethical standards of the responsible committee on human experimentation (institutional and national) and with the Helsinki Declaration of 1975, as revised in 2000. The study was approved by the Ethical Committee of the University of Szeged (139/2018-SZTE). The whole blood used for the preparation of the test samples originated from one of the authors (P.M.); therefore, no written consent was obtained. However, information on study characteristics and possible risks of blood sampling were verbally discussed among the authors beforehand and were documented in the internal protocol file.

### Sample preparation and instrumentation

Details of the sample preparation procedure have been reported previously [7]. Briefly, two spots 4.7 mm in diameter (corresponding to approx. 13.6 μl blood) of each of the DBS calibrators and QCs were extracted with freshly prepared IS working solution for 45 min at ambient temperature in 96-well microtiter plates shaken on a Heidolph Titramax 1000 plate shaker (Heidolph Instruments GmbH & CO. KG, Schwabach, Germany). The plate was sealed with a lid and shaken for 50 min at ambient temperature. Final concentrations were 0(0), 2(4), 5(10), 10(20), 25(50), 75(150) and 125(250) nM for steroids (Cort) in the DBS calibrators and 30(60) and 90(180) nM for steroids (Cort) in the QCs. The IS working solution (composition: 10 nM d_4_-Cort, 7.5 nM d_8_-21Deox and 1.5 nM each of d_2_-11Deox, d_5_-4AD and d_8_-17OHP) was prepared fresh daily by a 10-fold dilution of an intermediate solution mix of deuterated ISs with acetonitrile/water 80:20 (v/v). Following centrifugation for 10 min at 4°C and 1000 x g in an Eppendorf 5810R centrifuge (Eppendorf AG, Hamburg, Germany), the supernatant was transferred to a second microtiter plate and dried at 35°C in a gentle flow of air for 45 min. The residues were reconstituted in 45 μl of a methanol/water mixture (20:80 v/v).

Concentrations of Cort, 21Deox, 11Deox, 4AD and 17OHP were measured with an LC-MS/MS method with inter-day variabilities of approx. 10-15% that had previously been validated using clinical samples from CAH patients [7]. The system consisted of a PerkinElmer Flexar UHPLC system (solvent manager with a degasser, two FX-10 binary pumps, autosampler and thermostatic oven; all PerkinElmer Inc., Waltham, MA, USA), combined with an AB SCIEX QTRAP 5500 MS/MS triple quadrupole mass spectrometer, and controlled by Analyst 1.6.2 software (both AB SCIEX, Framingham, MA, USA). Chromatographic separation of the analytes was performed on a Phenomenex Kinetex XB-C18 100x3.0 mm, 2.6 μm core-shell analytical column and a SecurityGuard Ultra Cartridge guard column (both Phenomenex, Torrance, CA, USA). Eluent A consisted of ultrapure water (18.2 MΩ.cm, filtered through a 0.22 μm pore size membrane) plus 0.1% formic acid (LC-MS grade). Eluent B consisted of methanol plus 0.1% formic acid (both LC-MS grade). Results of the LC-MS/MS analysis are reported in nM.

### Statistical analysis

Statistical comparisons were performed by using the non-parametric Kruskal-Wallis test, followed by Dunn’s *post hoc* test (GraphPad Prism, GraphPad Software, La Jolla, CA, USA). Results are reported as medians (ranges). *p* values <0.05 were considered significant.

## Results

Inter-assay variations for Cort, 21Deox, 11Deox, 4AD and 17OHP at both the 30(60) nM and the 90(180) nM concentrations were generally within the predetermined ±15% range [16] for all five preparation approaches (S1 Table).

As concerns agreement with the nominal concentrations, the assayed values of the five steroids were within the acceptable ±15% range in case of four QC preparation approaches [16]. However, the decreased concentrations in Prep3 were outside of the ±15% range in 6 out of 10 comparisons (i.e. 5 analytes at 2 QC levels), being 11.7-31.2% lower than the nominal values in all 10 cases (S1 Table).

**Fig 1:**
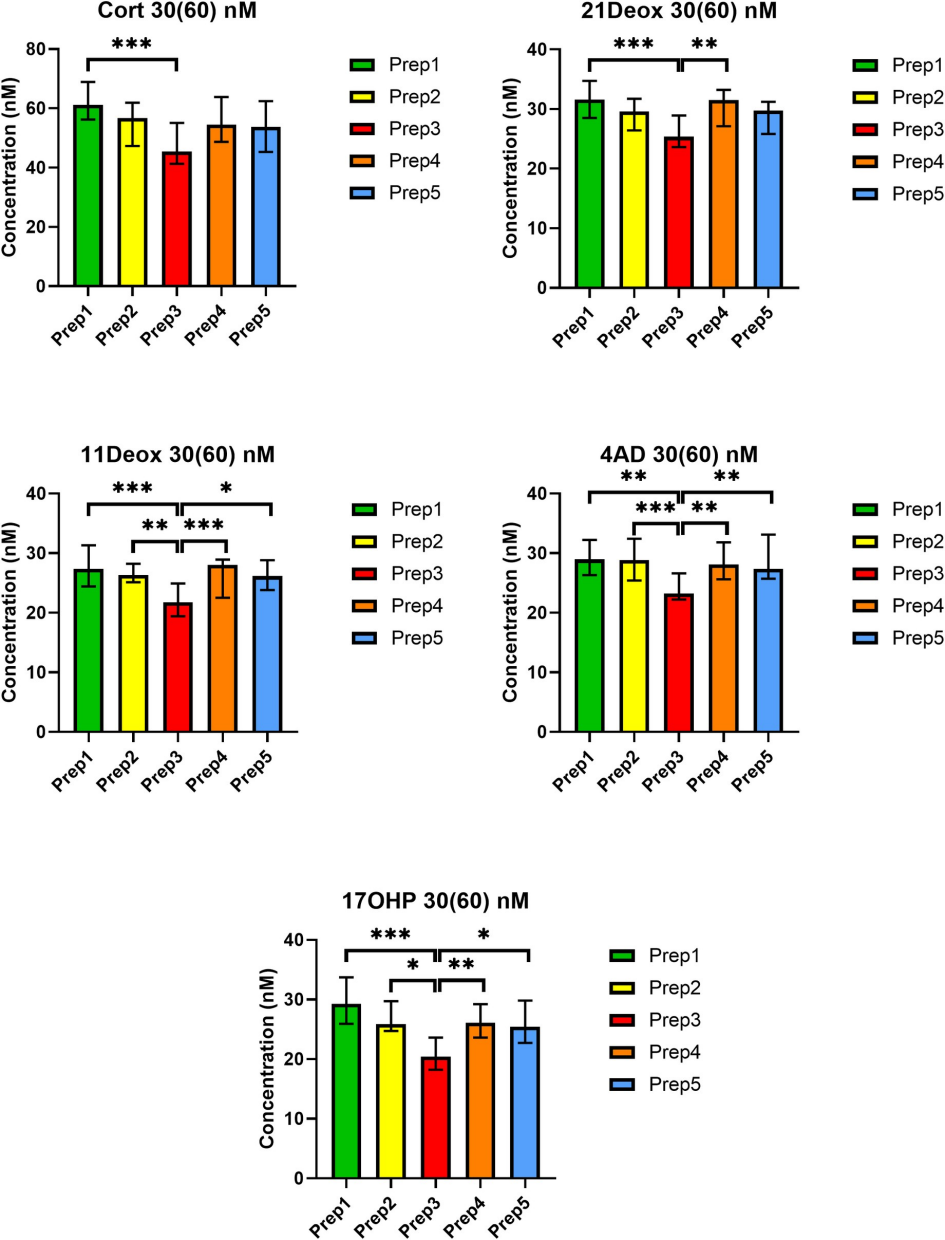
Comparison of steroid concentrations in five preparation procedures at QC level 30(60) nM. Values are given in nM as medians (ranges) for duplicates on five different days. Cort: cortisol; 21Deox: 21-deoxycortisol; 11Deox: 11-deoxycortisol; 4AD: 4-androstenedione; 17OHP: 17-hydroxyprogesterone. Non-parametric Kruskal-Wallis test and Dunn’s *post hoc* test. *** *p*<0.001, ** *p*<0.01 and * *p*<0.05 in the *post hoc* test.

**Fig 2:**
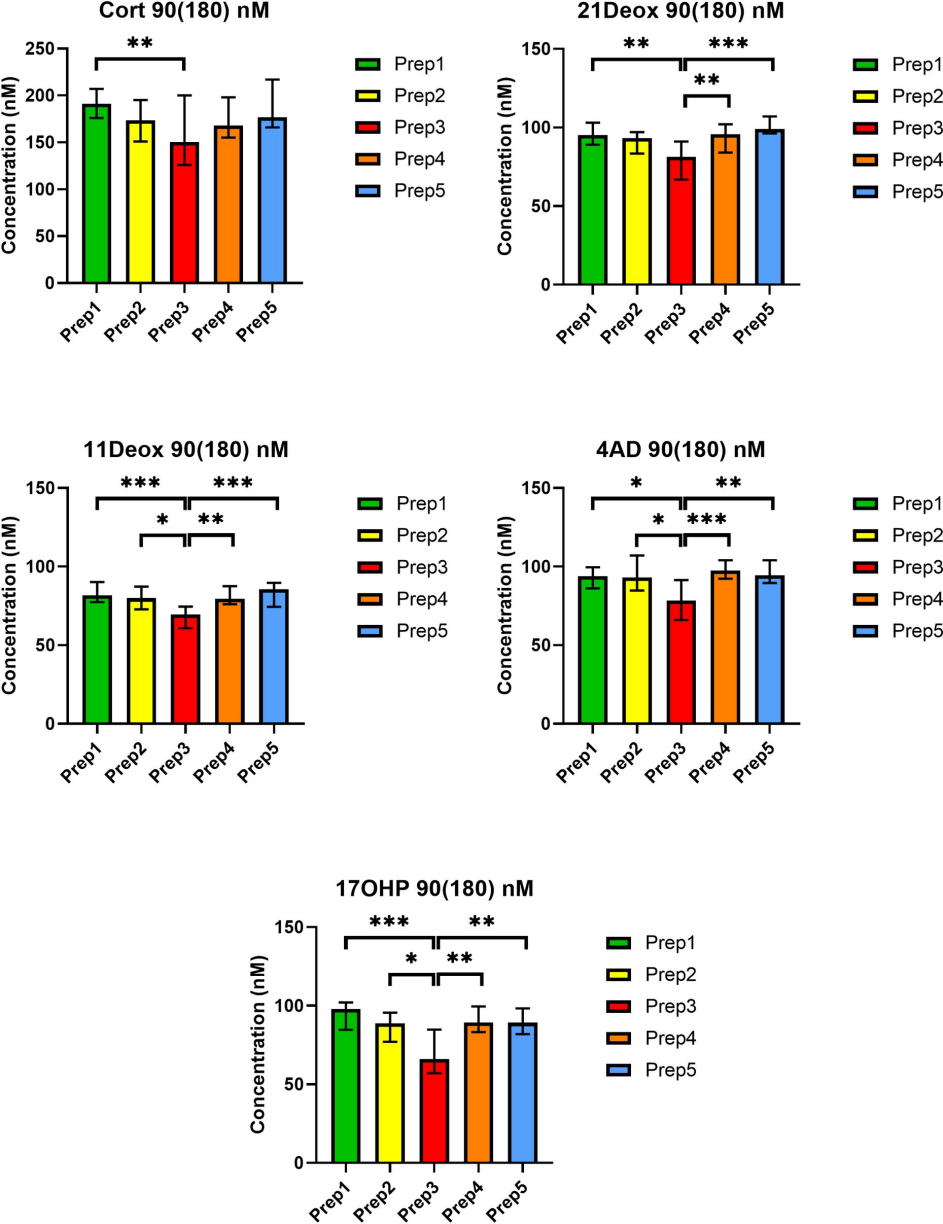
Comparison of steroid concentrations in five preparation procedures at QC level 90(180) nM. Values are given in nM as medians (ranges) for duplicates on five different days. Cort: cortisol; 21Deox: 21-deoxycortisol; 11Deox: 11-deoxycortisol; 4AD: 4-androstenedione; 17OHP: 17-hydroxyprogesterone. Non-parametric Kruskal-Wallis test and Dunn’s *post hoc* test. *** *p*<0.001, ** *p*<0.01 and * *p*<0.05 in the *post hoc* test.

Statistical analyses showed that the assayed concentrations were significantly different from each other for all five analytes at both QC levels (*p*<0.001, except for Cort at the 90(180) nM concentration where *p*<0.01). *Post hoc* analyses revealed that the statistical significance in the Kruskal-Wallis test was due to the decreased analyte concentrations for Prep3. In contrast, Prep1, Prep2, Prep4 and Prep5 did not differ significantly from each other for any of the analytes (*p*>0.05). Significance levels in the *post hoc* tests are depicted in Figs 1 and 2 and in the S1 Table.

## Discussion

A timely diagnosis and treatment can markedly decrease rates of serious morbidity and mortality associated with CAH [1, 2]. Accordingly, CAH is included in the NBS panel in numerous countries, including 24 European countries and all states of the USA [3]. Fluorimetric immunoassays are used in the NBS for CAH to detect elevations of 17OHP in DBS which, on the other hand, must be confirmed due to the high number of false-positives [1, 2]. The relatively low positive predictive value is attributed to factors that include 17OHP elevations due to factors other than CAH (stress, prematurity or sickness) and cross-reactions in the primary test [1, 17, 18]. Confirmation of the primary results is preferably performed using the same DBS specimen as in the primary screening (i.e. 2^nd^-tier testing) by means of LC-MS/MS [1, 2, 4].

These assays, similarly to other diagnostic methods, require daily QC samples for quality assurance purposes [4, 11]. However, even if 2^nd^-tier CAH testing QC material is available from the CDC, it is stated that: “*NSQAP QC materials are not a replacement for manufacturer kit controls or other daily QC*, *and should not be used for routine analysis*” [10].

According to reviews on applications of DBS such as therapeutic drug monitoring, the preparation technique can influence the quality of the DBS sample and consequently the accuracy of quantitation [11–13]. Factors include the sample collection technique, non-matrix spiking volume and solvent, homogeneity, technique of the application of blood onto filter paper and DBS storage [11–13]. However, DBS preparation in NBS is not covered in those recommendations. Issues with special emphasis in NBS include the need for a correct diagnostic interpretation (confirmation or exclusion of the raised diagnosis); the ability to measure a large number of samples with restricted turnaround time; and traceability and comparability of results both over extended periods and in international QC testing schemes.

Due to a lack of commercially available daily QCs in 2^nd^-tier CAH assays in NBS, various techniques have previously been utilized for the preparation of home-made QCs [6–8, 11]. In the protocol from Boelen and colleagues [6], red blood cells were washed three times with saline, mixed with steroid-depleted serum and spiked with steroid standard solutions. This approach (Prep1) is in close agreement with general protocols described in earlier reviews for non-NBS applications [11–13]. In the papers from Janzen *et al* [8] and Lacey and colleagues [9], the spiked samples were subsequently homogenized by means of freezing, prior to application onto filter cards. Further protocols applied two weeks of freezing [14] or three freeze-thaw cycles of the unspiked steroid-depleted whole blood, followed by spiking [7].

The present study is the first to evaluate five different QC preparation approaches used in the routine diagnostics for CAH for the 2^nd^-tier testing of Cort, 21Deox, 11Deox, 4AD and 17OHP in DBS. In terms of reproducibility, all five tested techniques (detailed in Table 1) gave similar and acceptable results, as shown by inter-assay variations <15% (S1 Table). Additionally, the assayed values of the tested steroids were also in good agreement with the nominal concentrations (within ±15%) in four of five preparation approaches (but not in Prep3); regardless of whether intact erythrocytes (Prep1 without freezing) or hemolyzed blood were present in the samples (Prep2, Prep4 and Prep5 with freezing). Statistical analyses confirmed the above findings, showing that the measured concentrations in Prep1, Prep2, Prep4 and Prep5 were similar for all analytes (*p*>0.05). Of note, analytes other than those tested here may give different results [11–13, 19].

In contrast, QCs prepared according to Prep3 (i.e. spiking followed by three freeze-thaw cycles) resulted in concentrations 11.7-31.2% lower than the nominal values in the present setting. In 6 out of 10 comparisons (i.e. 5 analytes at 2 QC levels), even the 15% limit [16] was not met (S1 Table). Results with Prep3 were also statistically significantly different from the other preparation approaches for all five analytes at both QC levels (Figs 1 and 2, S1 Table).

However, it should be noted that the observed statistical differences with Prep3 may not necessarily affect recognition of CAH patients negatively: based on the extent of the alterations, a correct interpretation may still be possible. Of note, concentrations of multiple steroids are determined in LC-MS/MS assays, allowing calculation of analyte ratios (precursor/product) which can markedly improve the diagnostic reliability of LC-MS/MS assays for CAH [1, 2, 4]. Thus, even if Preps 1, 2, 4 and 5 may seem most appropriate for the preparation of in-house QCs from the tested protocols, these results need to be confirmed in larger studies using additional sample preparation approaches.

The background of the observations of the present study is not fully understood but may be connected to possible roles of steroid-binding proteins [20], the effects of freeze-thaw cycles [10, 21] and other confounding factors. As an example, effects of the repeated freeze-thaw cycles are expected to be smaller if this procedure is performed on samples where analytes are almost absent (like in the steroid-depleted blood in Prep2) than for samples with higher analyte concentrations (e.g. with the already spiked blood in Prep3) [21]. This is in line with our findings for Prep2 and Prep3, differing only in the order of spiking and freezing. In contrast, Prep4 and Prep5 seemed similar, suggesting that the number of cycles may also play a role [21]. Further confounding factors are yet to be elucidated.

One could argue that freeze-thaw cycles and the resulting hemolysis of the blood have previously been suggested to be avoided in certain applications using DBSs including therapeutic drug monitoring [12, 13]. However, in the clinical setting, freeze-thaw cycles, as a technique to improve homogeneity and reproducibility of consecutive QC batches, have been used in several papers [7–9, 14], including the report from the CDC that provides worldwide quality assurance for diagnostic assays to facilitate a more reliable diagnosis of CAH and comparability of clinical laboratories [14].

Another limitation of the present study is that we did not examine long-term stability of each type of QCs. Storage stabilities could provide additional data for the comparison of the different preparation techniques. As an example, concentrations of Cort seem to decrease to a larger extent in Prep3 than in the other preparation approaches (S1 Table). Thus, stability assessment, similarly to that recently reported on Prep2 [22], may therefore be helpful in the decision. Moreover, a larger number of replicate analyses are needed for more accurate results.

Our experiments were planned with the aim to eliminate as many external sources of variance as possible. We used heparinized blood from a single person and single batches of phosphate-buffered saline and steroid-depleted serum for washing and mixing the red blood cells, respectively. Aliquots of the same steroid-depleted blood were then used in the five different QC preparation procedures (Table 1). Moreover, a single batch of standard solution was used for spiking all aliquots and the spiked whole blood was applied onto filter papers deriving from the same lot. A sample volume of 70 μl blood per circle allowed to punch four 4.7 mm spots from each blood circle in an „X” shape, each punch covering similar peripheral locations within a circle. To further eliminate potential variance from the analytical determination, the five types of QCs were assayed simultaneously with a single daily calibration row which was then used to calculate concentrations of all QCs on that day. Despite all efforts, it may not have been possible to eliminate all potential external factors.

Our findings have implications on the preparation of daily QCs in the clinical diagnostics for CAH. Even if external quality assurance like the one provided by the CDC remain essential [10, 14], the information presented here can facilitate a more reliable confirmation or exclusion of CAH in in-house methods. It may be speculated that our results on QCs may also be applicable for in-house prepared calibrators. Potential differences with various preparation techniques of home-made calibrators, similar to those found in QCs, could affect the reported concentrations and possibly the diagnostic performance of a CAH 2^nd^-tier assay [11–13]. However, this assumption is yet to be confirmed.

## Conclusions

For the first time, various QC preparation techniques used in the routine diagnostics for CAH, including Prep1 [6], an approach similar to general protocols reported for non-NBS applications of DBS [11–13], were compared using a validated LC-MS/MS method. For all five tested approaches, the reproducibility of the Cort, 21Deox, 11Deox, 4AD and 17OHP determination was shown to be similar and acceptable (i.e. <15%). The assayed values were also in good agreement with the nominal concentrations (within ±15%) in four techniques. However, Prep3 (i.e. spiking followed by three freeze-thaw cycles) resulted in significantly lower concentrations which in many cases exceeded the recommended ±15% limit. Of note, this statistical difference may not necessarily affect diagnostic accuracy negatively in recognizing CAH patients. These new findings provide additional information on in-house methods for CAH including 2^nd^-tier confirmatory assays in NBS. Even if the results of the present study suggest that Preps 1, 2, 4 and 5 seem most appropriate for the preparation of in-house QCs from the tested protocols, the observations need to be confirmed using additional sample preparation approaches with a larger number of replicate analyses, together with the assessment of long-term stability of the QC samples.

## Supporting information

S1 Fig (TIF)

S1 Table Details of the analytical results and statistical analyses. (XLSX)

## List of abbreviations

11Deox: 11-deoxycortisol
17OHP: 17-hydroxyprogesterone
21Deox: 21-deoxycortisol
4AD: 4-androstenedione
CAH: congenital adrenal hyperplasia
Cort: cortisol
DBS: dried blood spot
IS: internal standard
LC-MS/MS: liquid chromatography-tandem mass spectrometry
NBS: newborn screening
QC: quality control

## References

[1] El-Maouche D, Arlt W, Merke DP. Congenital adrenal hyperplasia. Lancet. 2017; 390: 2194–2210. 10.1016/S0140-6736(17)31431-9 28576284

[2] White PC. Neonatal screening for congenital adrenal hyperplasia. Nat Rev Endocrinol. 2009;5: 490–498. 10.1038/nrendo.2009.148 19690561

[3] Loeber JG, Platis D, Zetterström RH, Almashanu S, Boemer F, Bonham JR, et al. Neonatal Screening in Europe Revisited: An ISNS Perspective on the Current State and Developments Since 2010. Int J Neonatal Screen. 2021;7: 15. 10.3390/ijns7010015 33808002 PMC8006225

[4] Wooding KM, Auchus RJ. Mass spectrometry theory and application to adrenal diseases. Mol Cell Endocrinol. 2013;371: 201–207. 10.1016/j.mce.2012.12.026 23333773 PMC3625452

[5] Choi R, Park HD, Oh HJ, Lee K, Song J, Lee SY. Dried Blood Spot Multiplexed Steroid Profiling Using Liquid Chromatography Tandem Mass Spectrometry in Korean Neonates. Ann Lab Med. 2019;39: 263–270. 10.3343/alm.2019.39.3.263 30623618 PMC6340850

[6] Boelen A, Ruiter AFC, Claahsen-van der Grinten HL, Endert E, Ackermans MT. Determination of a steroid profile in heel prick blood using LC–MS/MS. Bioanalysis. 2016; 8:375–384. 10.4155/bio.16.6 26891684

[7] Monostori P, Szabó P, Marginean O, Bereczki C, Karg E. Concurrent Confirmation and Differential Diagnosis of Congenital Adrenal Hyperplasia from Dried Blood Spots: Application of a Second-Tier LC-MS/MS Assay in a Cross-Border Cooperation for Newborn Screening. Horm Res Paediatr. 2015;84: 311–318. 10.1159/000439380 26397944

[8] Janzen N, Sander S, Terhardt M, Steuerwald U, Peter M, Das AM, et al. Rapid steroid hormone quantification for congenital adrenal hyperplasia (CAH) in dried blood spots using UPLC liquid chromatography-tandem mass spectrometry. Steroids. 2011;76: 1437–1442. 10.1016/j.steroids.2011.07.013 21839763

[9] Lacey JM, Minutti CZ, Magera MJ, Tauscher AL, Casetta B, McCann M, et al. Improved specificity of newborn screening for congenital adrenal hyperplasia by second-tier steroid profiling using tandem mass spectrometry. Clin Chem. 2004;50: 621–625. 10.1373/clinchem.2003.027193 14656905

[10] CDC 2020 Set1 Quality Control Report. Available from: https://www.cdc.gov/labstandards/nsqap_reports.html (Accessed: April 1^st^ 2021).

[11] Wagner M, Tonoli D, Varesio E, Hopfgartner G. The use of mass spectrometry to analyze dried blood spots. Mass Spectrom Rev. 2016;35: 361–438. 10.1002/mas.21441 25252132

[12] Enderle Y, Foerster K, Burhenne J. Clinical feasibility of dried blood spots: Analytics, validation, and applications. J Pharm Biomed Anal. 2016;130: 231–243. 10.1016/j.jpba.2016.06.026 27390013

[13] Edelbroek PM, van der Heijden J, Stolk LM. Dried blood spot methods in therapeutic drug monitoring: methods, assays, and pitfalls. Ther Drug Monit. 2009;31: 327–336. 10.1097/FTD.0b013e31819e91ce 19349929

[14] De Jesús VR, Mei JV, Cordovado SK, Cuthbert CD. The Newborn Screening Quality Assurance Program at the Centers for Disease Control and Prevention: Thirty-five Year Experience Assuring Newborn Screening Laboratory Quality. Int J Neonatal Screen. 2015;1: 13–26. 10.3390/ijns1010013 26309908 PMC4545740

[15] Koster RA, Alffenaar JW, Botma R, Greijdanus B, Touw DJ, Uges DR, et al. What is the right blood hematocrit preparation procedure for standards and quality control samples for dried blood spot analysis? Bioanalysis. 2015;7: 345–351. 10.4155/bio.14.298 25697192

[16] Guideline on bioanalytical method validation. European Medicines Agency Reference number: EMEA/CHMP/EWP/192217/2009 Rev. 1 Corr. 2. Effective from: February 1^st^ 2012. Available from: https://www.ema.europa.eu/en/bioanalytical-method-validation (Accessed: April 1st 2021).

[17] Kamrath C, Hartmann MF, Boettcher C, Wudy SA. Reduced activity of 11β-hydroxylase accounts for elevated 17α-hydroxyprogesterone in preterms. J Pediatr. 2014;165: 280–284. 10.1016/j.jpeds.2014.04.011 24862381

[18] al Saedi S, Dean H, Dent W, Stockl E, Cronin C. Screening for congenital adrenal hyperplasia: the Delfia Screening Test overestimates serum 17-hydroxyprogesterone in preterm infants. Pediatrics. 1996;97: 100–102. 8545200

[19] Hoogtanders K, van der Heijden J, Christiaans M, Edelbroek P, van Hooff JP, Stolk LM. Therapeutic drug monitoring of tacrolimus with the dried blood spot method. J Pharm Biomed Anal. 2007;44: 658–664. 10.1016/j.jpba.2006.11.023 17184953

[20] Hammond GL. Plasma steroid-binding proteins: primary gatekeepers of steroid hormone action. J Endocrinol. 2016;230: R13–25. 10.1530/JOE-16-0070 27113851 PMC5064763

[21] González-Domínguez R, González-Domínguez Á, Sayago A, Fernández-Recamales Á. Recommendations and Best Practices for Standardizing the Pre-Analytical Processing of Blood and Urine Samples in Metabolomics. Metabolites. 2020;10: 229. 10.3390/metabo10060229 32503183 PMC7344701

[22] Grecsó N, Zádori A, Szécsi I, Baráth Á, Galla Z, Bereczki C, et al. Storage stability of five steroids and in dried blood spots for newborn screening and retrospective diagnosis of congenital adrenal hyperplasia. PLoS One. 2020;15: e0233724. 10.1371/journal.pone.0233724 32470014 PMC7259505

