# Supplementary figures and images for: Comparison of different preparation techniques of dried blood spot quality controls in newborn screening for congenital adrenal hyperplasia
Source: PLoS One. 2021 May 20;16(5):e0252091. doi: 10.1371/journal.pone.0252091 (PMC8136632; doi:10.1371/journal.pone.0252091)

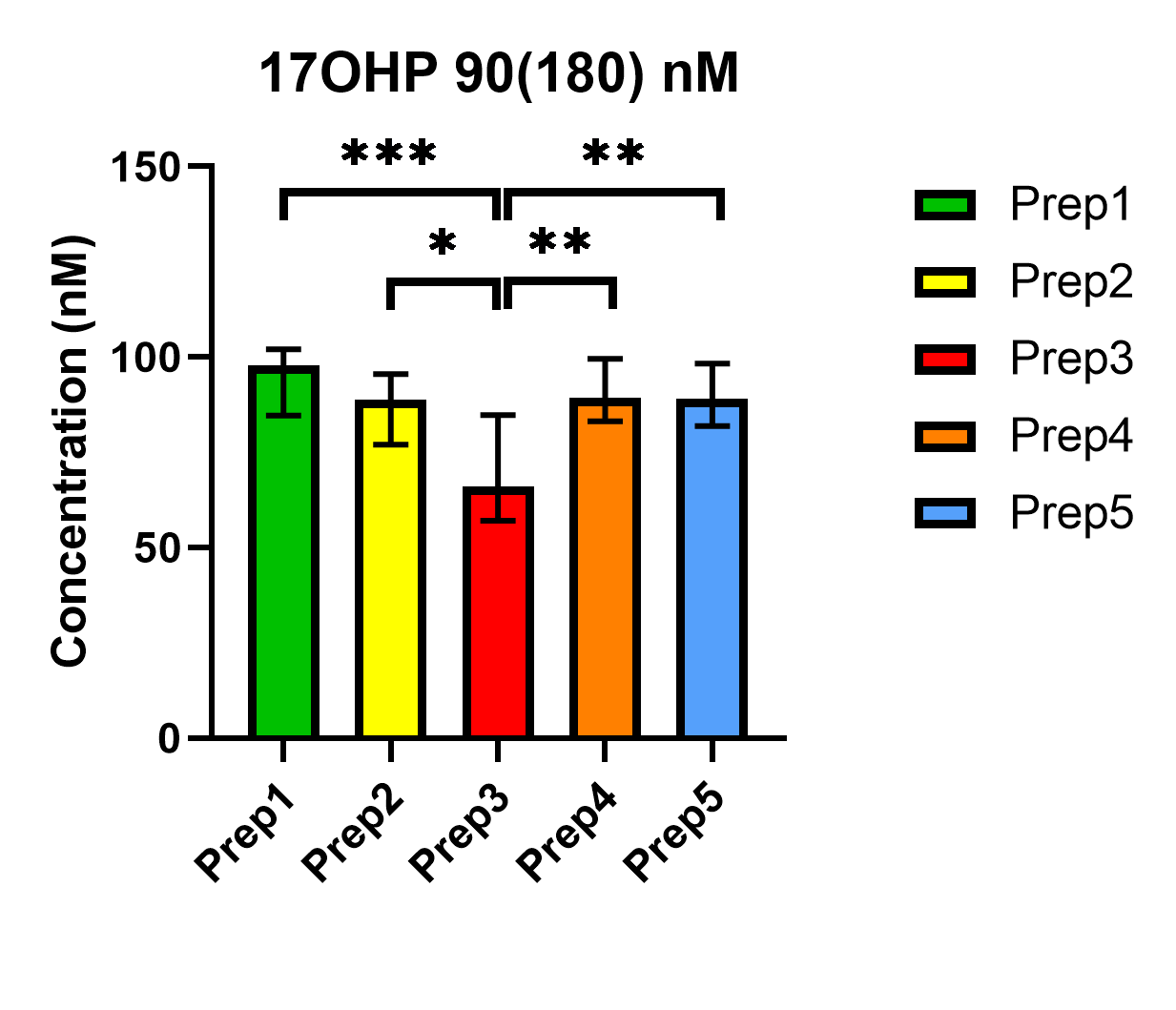

Supplement: S1 Fig — (TIF) [file pone.0252091.s001.tif]
